# Impact of Pd Loading on CO_2_ Reduction Performance over Pd/TiO_2_ with H_2_ and H_2_O

**DOI:** 10.3390/molecules25061468

**Published:** 2020-03-24

**Authors:** Akira Nishimura, Tadaaki Inoue, Yoshito Sakakibara, Masafumi Hirota, Akira Koshio, Eric Hu

**Affiliations:** 1 Division of Mechanical Engineering, Graduate School of Engineering, Mie University, Tsu, Mie 514-8507, Japan; 418M109@m.mie-u.ac.jp (T.I.); 419M117@m.mie-u.ac.jp (Y.S.); hirota@mach.mie-u.ac.jp (M.H.); 2 Division of Chemistry for Materials, Graduate School of Engineering, Mie University, Tsu, Mie 514-8507, Japan; koshio@chem.mie-u.ac.jp; 3 School of Mechanical Engineering, the University of Adelaide, SA 5005, Australia; eric.hu@adelaide.edu.au

**Keywords:** Pd/TiO_2_ photocatalyst, CO_2_ reduction, Pd loading weight, combination of reductants

## Abstract

This study investigated the impact of molar ratio of CO_2_ to reductants H_2_O and H_2_, as well as Pd loading weight on CO_2_ reduction performance with Pd/TiO_2_ as the photocatalyst. The Pd/TiO_2_ film photocatalyst is prepared by the sol-gel and dip-coating process to prepare TiO_2_ film and the pulse arc plasma method is used to dope Pd on TiO_2_ film. The prepared Pd/TiO_2_ film was characterized by SEM, EPMA, STEM, EDS, and EELS. This study also investigated the performance of CO_2_ reduction under the illumination condition of Xe lamp with or without ultraviolet (UV) light. As a result, it is revealed that when the molar ratio of CO_2_/H_2_/H_2_O is set at 1:0.5:0.5, the best CO_2_ reduction performance has been obtained under the illumination condition of Xe lamp with and without UV light. In addition, it is found that the optimum Pd loading weight is 3.90 wt%. The maximum molar quantities of CO and CH_4_ produced per unit weight of photocatalyst are 30.3 μmol/g and 22.1 μmol/g, respectively, for the molar ratio of CO_2_/H_2_/H_2_O = 1:0.5:0.5 under the condition of Xe lamp illumination with UV light. With UV light, C_2_H_4_ and C_2_H_6_, as well as CO and CH_4_ are also produced by the Pd/TiO_2_ film photocatalyst prepared in this study.

## 1. Introduction

The Paris Agreement adopted in 2015 set the goal that the increase in average temperature in the world from the industrial revolution by 2030 should be kept less than 2 K. However, the global mean concentration of CO_2_ in the atmosphere has increased up to 410 ppmV in December 2019, which increased by 25 ppmV since 2009 [1]. Therefore, it is requested to develop a new CO_2_ reduction/utilization technology in order to reduce the amount of CO_2_ in the atmosphere.

Reducing or converting CO_2_ into fuel by photocatalyst became a hot R&D area. TiO_2_ is commonly used as a photocatalyst for CO_2_ reduction since it is convenient, inexpensive, and has strong durability for chemicals and corrosion [2]. TiO_2_ is a popular photocatalyst that can reduce CO_2_ into CO, CH_4_, CH_3_OH, and H_2_ etc. with ultraviolet (UV) light [3,4,5].

Since pure TiO_2_ can only be activated under UV light illumination, it is not very effective under sunlight illumination as UV light accounts for only approximately 4% in the solar spectrum. In addition, the rate of electron/hole pair recombination is faster than the rate of chemical interaction between the absorbents during redox reactions when using pure TiO_2_ [6].

Many attempts have been reported to improve the performance of the TiO_2_ [3]. Doping precious metals such as Pt [7], Ag [8], Au [9], Cu [10,11], using composite materials formed by GaP and TiO_2_ [12], combining CdS/TiO_2_ in order to utilize two photocatalysts that have different band gaps [13], adding carbon-based AgBr nanocomposites into TiO_2_ [14], sensitizing CuInS_2_ and TiO_2_ hybrid nanofibers [15], and preparing a procedure of TiO_2_ using two alcohols (ethanol and isopropyl alcohol) and supercritical CO_2_ [16] are some of the attempts to promote the performance of TiO_2_. Though the CO_2_ reduction performance was improved to a certain degree in these attempts, the concentrations of the products were still low, which were ranging from 1 to 150 μmol/g-cat [7,8,9,10,11,12,13,14,15,16].

Among various metals that have been used for doping, Pd is considered as a favorite candidate [17,18,19], since Pd can extend the absorption band to 400–800 nm [20,21], which covers the whole visible light range. Pd/TiO_2_ performs a higher reduction performance compared to pure TiO_2_, especially, to produce hydrocarbon [20,21,22]. In addition, it is known that the CO_2_ reduction performance of Pd/TiO_2_ is superior to that of TiO_2_ from the viewpoint of producing CH_4_ and H_2_ [7,19]. This is due to the work function of Pd, which reflects the electron donating or accepting ability. In addition, it is thought that Pd loaded on TiO_2_ functions to increase the efficiency of photogenerated electrons for the formation of reductive products.

According to the literature survey, H_2_O or H_2_ were normally used as the reductants for CO_2_ reduction over Pd/TiO_2_ [17,18,19,20,21,22,23]. In studies of CO_2_ reduction with H_2_O [17,18,19,20,21,22], the mixture ratio of CO_2_ and H_2_O was fixed. According to the report on CO_2_ reduction with H_2_ [23], the molar ratio of CO_2_:H_2_ was fixed at 1:4, but the impact of the ratio on CO_2_ reduction performance of Pd/TiO_2_ was not investigated. Though it is thought that the mixture ratio of CO_2_ and reductants influences the CO_2_ reduction performance of Pd/TiO_2_, there was no other study investigating it nor the effect of using both H_2_O and H_2_ as reductants on CO_2_ reduction over Pd/TiO_2_ except the study conducted by the authors [24]. In addition, the metal loading weight with TiO_2_ is important to improve the CO_2_ reduction performance [25,26]. However, there was no study so far to qualify the improvement.

To promote the CO_2_ reduction performance, the optimum reductant providing the proton (H^+^) should be clarified. According to the previous studies [27,28,29,30], the reaction mechanism to reduce CO_2_ with H_2_O can be summarized as shown below:

<Photocatalytic reaction>

TiO_2_ + *hν* → *h*^+^ + *e*^−^ (1)

<Oxidization>

2H_2_O + 4*h*^+^ → 4H^+^ + O_2_ (2)

<Reduction>

CO_2_ + 2H^+^ + 2*e*^−^ → CO + H_2_O (3)

CO_2_ + 8H^+^ + 8*e*^−^ → CH_4_ + 2H_2_O (4)

2CO_2_ + 12H^+^ + 12*e*^−^ → C_2_H_4_ + 4H_2_O (5)

2CO_2_ + 14H^+^ + 14*e*^−^ → C_2_H_6_ + 4H_2_O (6)

The reaction mechanism to reduce CO_2_ with H_2_ can be summarized as shown below [30,31].

<Photocatalytic reaction>

TiO_2_ + *hν* → *h*^+^ + *e*^−^ (7)

<Oxidization>

H_2_ → 2H^+^ + 2*e*^−^ (8)

<Reduction>

CO_2_ + *e*^−^ → CO_2_^−^ (9)

CO_2_^−^+ H^+^ +*e*^−^ → HCOO^−^ (10)

HCOO^−^ + H^+^ → CO + H_2_O (11)

CO_2_ + 8*e*^−^ + 8H^+^ → CH_4_ + 2H_2_O (12)

2CO_2_ + 12*e*^−^ + 12H^+^ → C_2_H_4_ + 4H_2_O (13)

2CO_2_ + 14*e*^−^ + 14H^+^ → C_2_H_6_ + 4H_2_O (14)

Though a few studies using pure TiO_2_ under CO_2_/H_2_/H_2_O condition were reported [32,33,34], the effect of ratio of CO_2_, H_2_, and H_2_O, as well as the effect of Pd loading on CO_2_ reduction characteristics was not investigated previously.

The purpose of this study is to clarify the effect of molar ratio of CO_2_ to reductants of H_2_ and H_2_O on CO_2_ reduction characteristics with Pd/TiO_2_. Additionally, the present study also aims to clarify the optimum combination of reductants, as well as Pd loading weight with TiO_2_.

The present study employed TiO_2_ films coated on netlike glass fibers (SILIGLASS U, Nihonmuki Co., Tokyo, Japan) by the sol-gel and dip-coating process. The glass fiber whose diameter is about 10 μm is weaved as a net, resulting in the diameter of collected fiber of approximately 1 mm. As to the specification of each fiber, the porous diameter is approximately 1 nm and the specific surface area is approximately 400 m^2^/g. The composition of netlike glass fiber is SiO_2_ of 96 wt%. The aperture area is approximately 2 mm × 2 mm. Due to the porous structure of the netlike glass fiber, the TiO_2_ film can be captured on netlike glass fiber easily in the step of preparation by sol-gel and dip-coating procedure. Additionally, it was believed that CO_2_ would be more easily absorbed by the prepared photocatalyst since the porous fiber has a large surface area [35,36].

After the coating of TiO_2_, nanosized Pd particles were loaded on TiO_2_ by the pulse arc plasma method applying high voltage. The pulse number can be controlled by the quantity of Pd loaded. The Pd loading weight on TiO_2_ was measured by Electron Probe Micro Analyzer (EPMA).

In this paper, the characterization of Pd/TiO_2_ was conducted by Scanning Electron Microscope (SEM), EPMA, Scanning Transmission Electron Microscope (STEM), Energy Dispersive X-ray Spectrometer (EDS), and Electron Energy Loss Spectrum (EELS) analysis before the CO_2_ reduction experiment. The performances of CO_2_ reduction with H_2_ and H_2_O under the condition of illuminating Xe lamp including or excluding UV light were investigated in this paper. The combination of CO_2_/H_2_/H_2_O was changed for 1:0.5:0.5, 1:0.5:1, 1:1:0.5, 1:1:1, and 1:2:2 based on molar ratio to clarify the optimum combination of CO_2_/H_2_/H_2_O for CO_2_ reduction with Pd/TiO_2_. If the amount of H_2_ is larger than that of H_2_O, it is thought that the effect of H_2_O on the photocatalytic reaction is higher. On the other hand, if the amount of H_2_O is larger than that of H_2_, it is thought that the effect of H_2_O on the photocatalytic reaction is higher. This study investigated the effect of H_2_ or H_2_O on the CO_2_ reduction performance of Pd/TiO_2_ under the condition of CO_2_/H_2_/H_2_O for the first time, so the originality of this study could be justified. In addition, the effect of Pd loading weight with TiO_2_ on CO_2_ reduction performance was also investigated in this study.

## 2. Results and Discussion

### 2.1. Characterization Analysis of Pd/TiO_2_ Film

**Figure 1:**
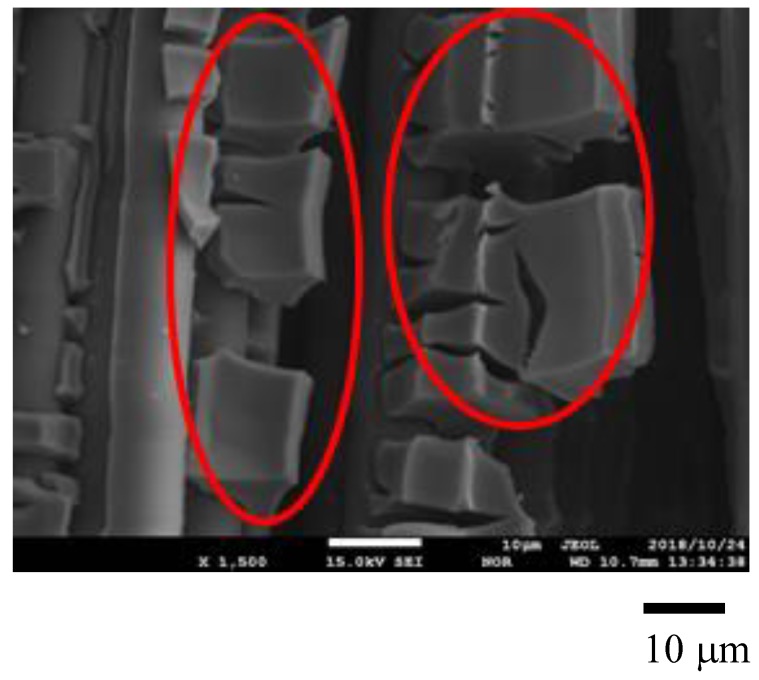
SEM result of TiO_2_ film coated on netlike glass disc.

**Figure 2:**
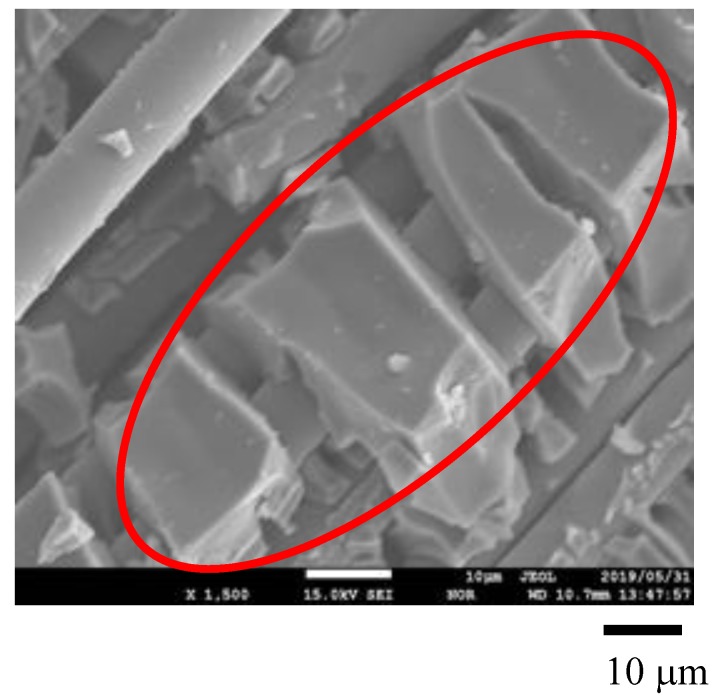
SEM result of Pd/TiO_2_ film coated on netlike glass disc.

**Figure 3:**
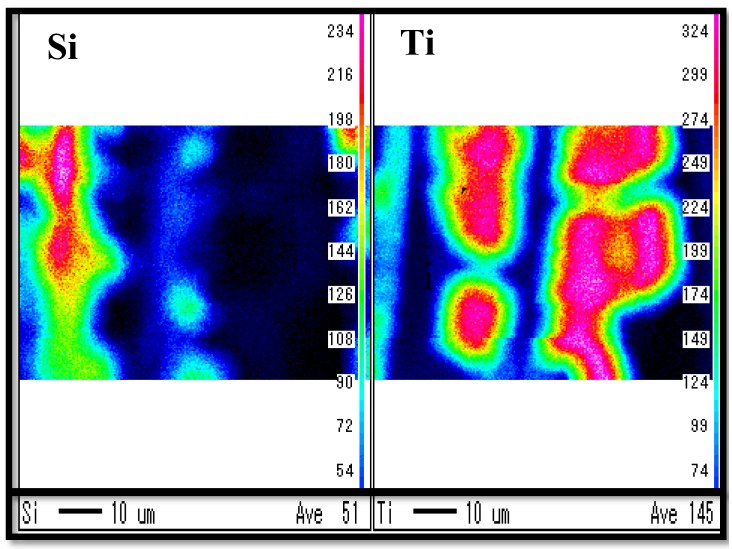
EPMA result of TiO_2_ film coated on netlike glass disc.

**Figure 4:**
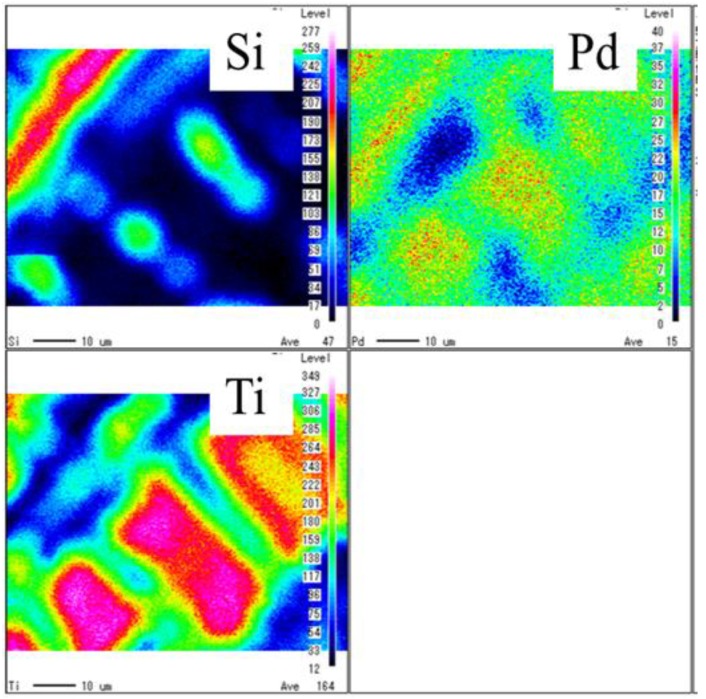
EPMA result of Pd/TiO_2_ film coated on netlike glass disc.

Figure 1, Figure 2 show SEM images of TiO_2_ film and Pd/TiO_2_ film coated on netlike glass disc, respectively. The SEM images were taken at 1500 times magnification. In these figures, the red circles indicate TiO_2_ according to EPMA results. Figure 3 and Figure 4 show EPMA results of TiO_2_ and Pd/TiO_2_ film coated on netlike glass disc, respectively. The data with the weight percentage of Pd to Pd/TiO_2_ film of 4.97 wt% are shown in Figure 4 as an example. In these figures, the different colors indicate the concentrations of each element in the observation area. For example, light colors such as white, pink, and red mean the quantity of element is small.

According to these figures, it is clear that TiO_2_ film was coated on netlike glass fiber. In addition, it is observed that the crack is formed on the TiO_2_ film. Since the thermal conductivity is different between Ti and SiO_2_, which are 19.4 W/(m K) and 1.82 W/(m K), respectively at 600 K [37], the temperature distribution of TiO_2_ solution adhered on the netlike glass disc was not uniform during the firing process, as a result, cracks were formed on the TiO_2_ film by the thermal expansion and shrinkage around netlike glass fiber. As to the crystal structure of TiO_2_, it is thought to be anatase since the firing temperature was set at 623 K in this study. A previous study [38] found the crystal structure of prepared TiO_2_ was anatase if the firing temperature was from 673 K to 873 K, while it would be rutile if the firing temperature was 973 K. The uniform loading of nanosized Pd particles on TiO_2_ was observed according to Figure 3.

The observation area of diameter of 300 μm is analyzed by EPMA to evaluate the quantity of loaded Pd within TiO_2_ film. Twenty observation points obtained from several samples were used to determine the weight percentages of Pd and Ti in this study. As a result, the weight percentages of Pd to Pd/TiO_2_ film prepared by changing pulse number in this study are 0.49 wt%, 3.90 wt%, and 4.97 wt%, while the weight percentage of Ti are 99.51 wt%, 96.10 wt%, and 95.03 wt%, respectively.

**Figure 5:**
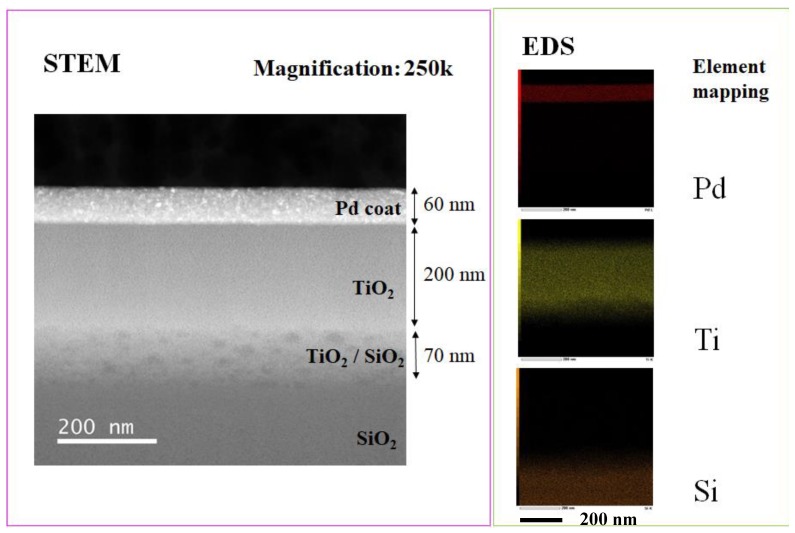
STEM and EDS analysis result of Pd/TiO_2_ film coated on netlike glass disc.

Figure 5 shows STEM and EDS results of Pd/TiO_2_ film coated on netlike glass disc. 250,000 times magnification STEM image was used for the EDS analysis. It is observed that Pd is coated on TiO_2_ film according to STEM image, which is confirmed from EDS image. It is also observed that the layout of Pd and Ti are separated. The thickness of the Pd coated is approximately 60 nm. Nanosized Pd particles are loaded on TiO_2_ dispersedly.

**Figure 6:**
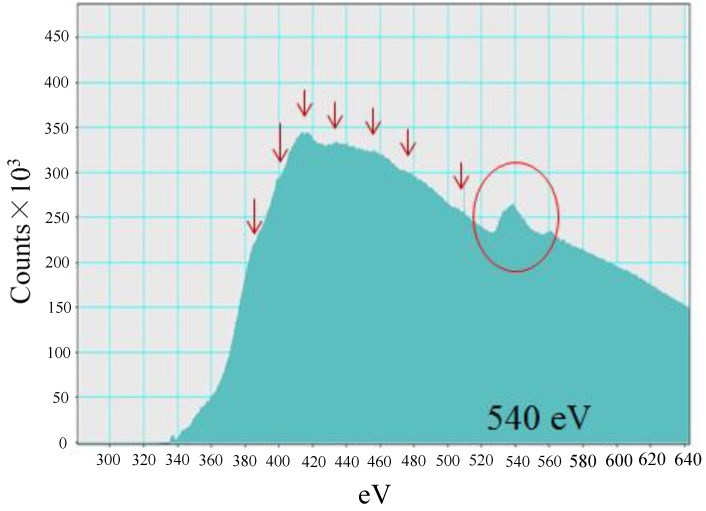
EELS spectra of Pd in Pd/TiO_2_.

Figure 6 shows EELS spectra of Pd in Pd/TiO_2_ film which peaks at around 540 eV. Comparing the spectra peaks of Pd nanowire with that of Pd metal and PdO in [39], the EELS spectra of Pd metal matches that in Figure 6. Therefore, it is believed that the Pd in Pd/TiO_2_ prepared in this study exists as Pd metal. Since the photoreduction performance of Pd/TiO_2_ was higher than that of PdO/TiO_2_ [40,41], the desirable Pd/TiO_2_ without oxidization was proved to be prepared in this study.

### 2.2. Impact of Molar Ratio of CO_2_, H_2_, and H_2_O, as well as Pd Loading Weight on CO_2_ Reduction Performance

**Figure 7:**
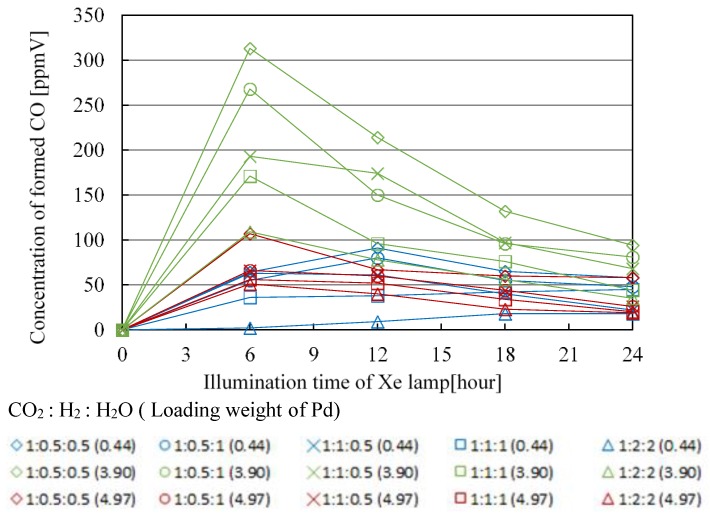
Change in concentration of formed CO with the illumination time among different molar ratios of CO_2_/H_2_/H_2_O and Pd loading weight with ultraviolet (UV) light illumination.

**Figure 8:**
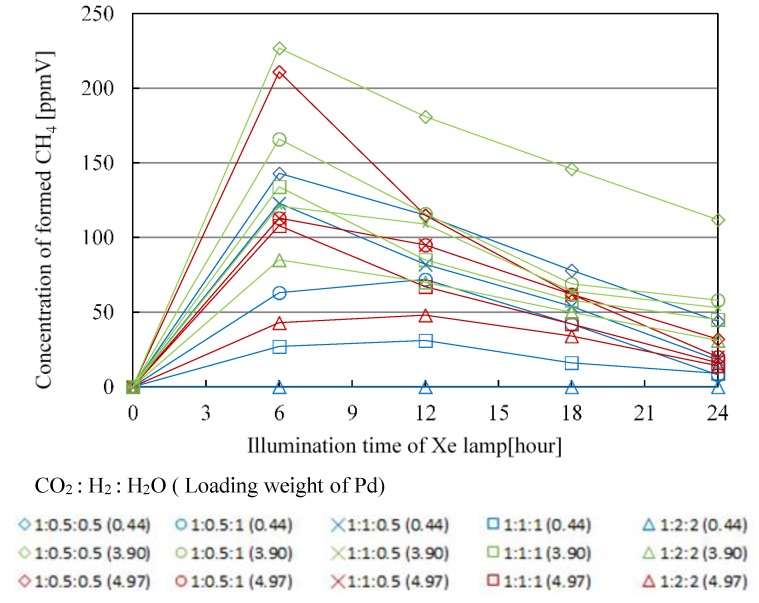
Change in concentration of formed CH_4_ with the illumination time among different molar ratios of CO_2_/H_2_/H_2_O and Pd loading weight with UV light illumination.

**Figure 9:**
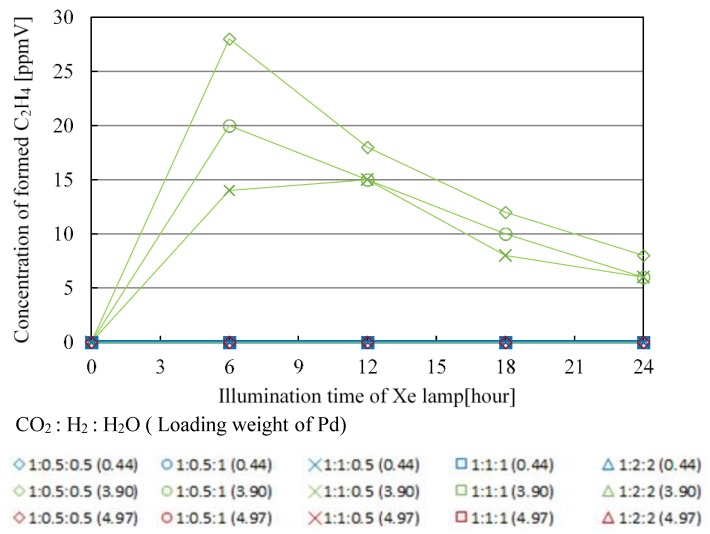
Change in concentration of formed C_2_H_4_ with the illumination time among different molar ratios of CO_2_/H_2_/H_2_O and Pd loading weight with UV light illumination.

**Figure 10:**
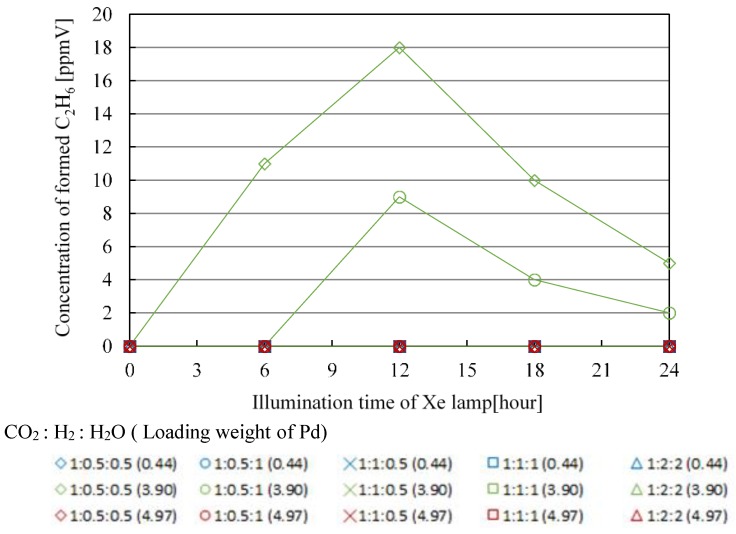
Change in concentration of formed C_2_H_6_ with the illumination time among different molar ratios of CO_2_/H_2_/H_2_O and Pd loading weight with UV light illumination.

Figure 7, Figure 8, Figure 9 and Figure 10 show the change in concentration of formed CO, CH_4_, C_2_H_4_, and C_2_H_6_ with Pd/TiO_2_ film coated on netlike glass disc with the time under the condition of Xe lamp illumination with UV light, respectively. In these figures, the impact of molar ratio of CO_2_, H_2_, and H_2_O, as well as Pd loading weight are also presented. Before this experiment, a blank test without Xe lamp illumination had been carried out as a reference, resulting that no fuel was detected as expected. Table 1, Table 2, Table 3 and Table 4 list the maximum concentration of formed CO, CH_4_, C_2_H_4_, and C_2_H_6_ under the condition shown in Figure 7, Figure 8, Figure 9 and Figure 10, respectively.

According to Figure 7, Figure 8, Figure 9 and Figure 10 and Table 1, Table 2, Table 3 and Table 4, the CO_2_ reduction performance to produce CO, CH_4_, C_2_H_4_, and C_2_H_6_ is the highest at the molar ratio of CO_2_/H_2_/H_2_O = 1:0.5:0.5. Since the reaction scheme of CO_2_/H_2_/H_2_O has not been fully understood, Equations (1)–(15) are used to explain the results. Equations (1)–(15) show that the theoretical molar ratio of CO_2_ with H_2_O or H_2_ to produce CO is 1:1. On the other hand, the theoretical molar ratio of CO_2_ with H_2_O or H_2_ to produce CH_4_ is 1:4. In addition, CH_4_, C_2_H_4_, and C_2_H_6_ are produced in the series after CO is produced. For example, producing CH_4_ needs four times H^+^ and electrons as many as producing CO needs. The other fuels such as C_2_H_4_ and C_2_H_6_ need more H^+^ and electrons compared to producing CH_4_. Since Pd has a high reduction performance [21,22,40], it is thought that the optimum molar ratio of CO_2_/total reductants to produce CH_4_, C_2_H_4_, and C_2_H_6_ is smaller than the theoretical molar ratio required. Moreover, since the molar ratio of H_2_ is the same as that of H_2_O under the molar ratio of CO_2_/H_2_/H_2_O = 1:0.5:0.5 condition, the effect of H_2_ or H_2_O is not higher than that of the other to obtain the optimum molar ratio of CO_2_/H_2_/H_2_O over Pd/TiO_2_ photocatalyst. However, according to Table 1, Table 2, Table 3 and Table 4, the CO_2_ reduction performance for the condition that the molar ratio of H_2_O is larger than that of H_2_ is better, resulting in that the effect of H_2_O is bigger than that of H_2_ to promote the CO_2_ reduction performance over Pd/TiO_2_ totally in this study.

In addition, it is known from Figure 7, Figure 8, Figure 9 and Figure 10 and Table 1, Table 2, Table 3 and Table 4 that the maximum concentration of produced fuel is obtained when Pd loading weight is 3.90 wt% irrespective of fuel type. One might think that the CO_2_ reduction performance is promoted with increasing Pd loading weight. However, it is believed that too much Pd loading causes covering the surface of TiO_2_ film [42,43], resulting in that CO_2_ and reductants cannot attain the surface of TiO_2_ film sufficiently. Consequently, it is clear that there is an optimum Pd loading weight to promote CO_2_ reduction performance with H_2_ and H_2_O.

Table 5, Table 6, Table 7 and Table 8 list the maximum molar quantities of CO, CH_4_, C_2_H_4_, and C_2_H_6_ per unit weight of photocatalyst under the condition of Xe lamp illumination with UV light, respectively. The quantities of Pd/TiO_2_ coated on netlike glass disc for Pd loading weight of 0.44 wt%, 3.90 wt%, and 4.97 wt% are 0.05 g, 0.05 g, and 0.09 g, respectively. These quantities of Pd/TiO_2_ coated on netlike glass disc were measured by an electric balance comparing the weights of several samples before and after preparing Pd/TiO_2_ film on netlike glass fiber. The photocatalytic activity evaluation using molar quantities of product per weight of photocatalyst was adopted as in the recent photocatalyst studies [44,45,46,47].

**Figure 11:**
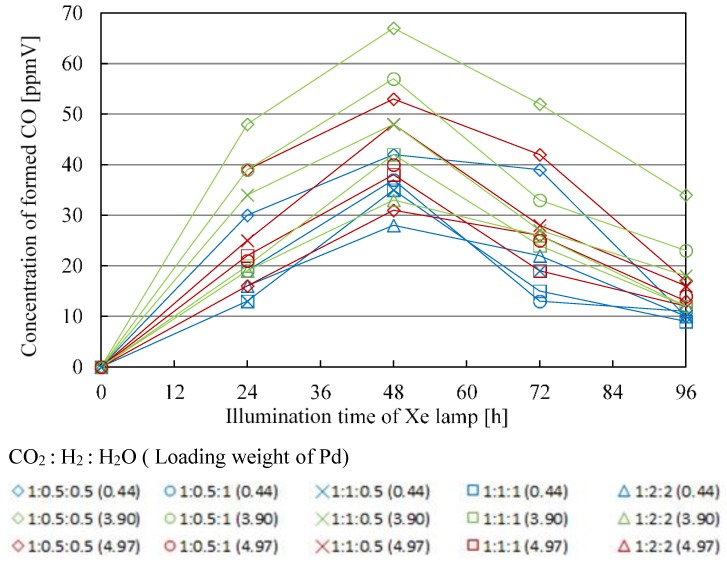
Change in concentration of formed CO with the illumination time among different molar ratios of CO_2_/H_2_/H_2_O and Pd loading weight without UV light illumination.

**Figure 12:**
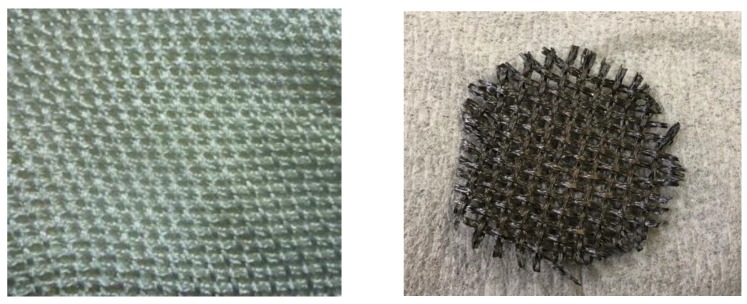
Photos of netlike glass disc before and after coating of Pd/TiO_2_ (left: Before; right: After).

**Figure 13:**
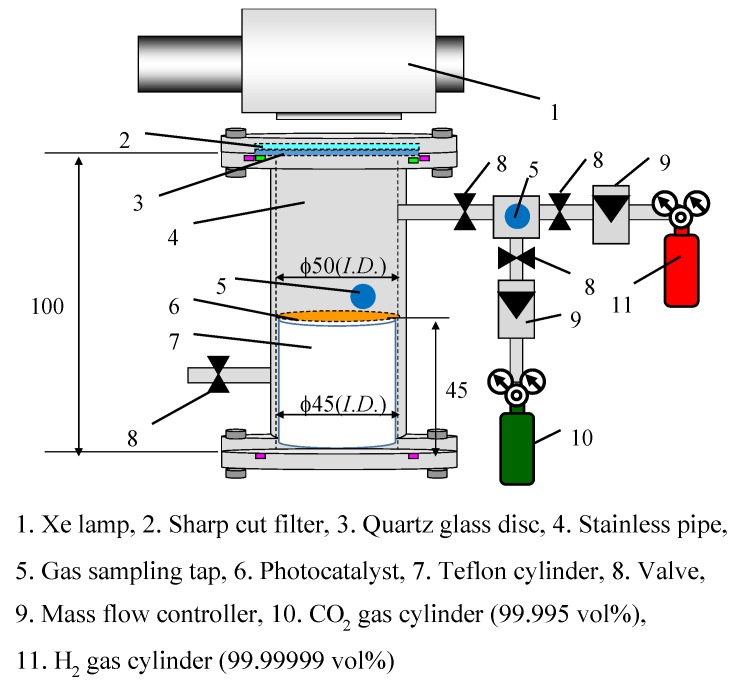
Experimental setup for CO_2_ reduction [49,50].

**Figure 14:**
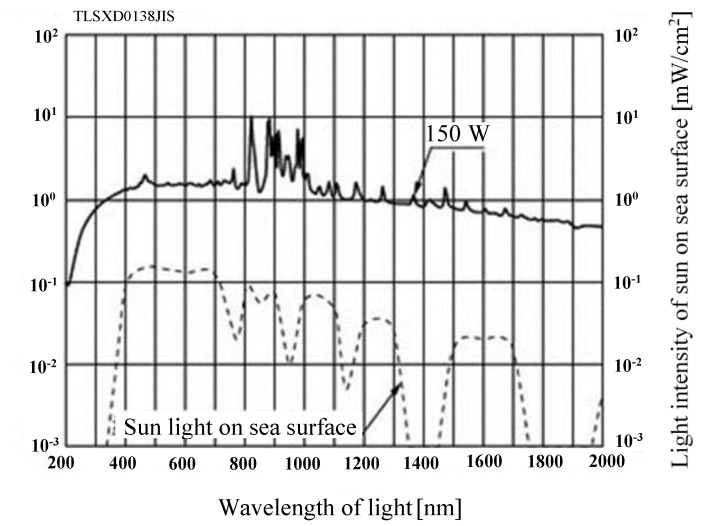
Spectra data on light intensity of Xe lamp without edge filter.

According to Table 5, Table 6, Table 7 and Table 8, the maximum molar quantities of CO, CH_4_, C_2_H_4_, and C_2_H_6_ per unit weight of photocatalyst are obtained for the molar ratio of CO_2_/H_2_/H_2_O = 1:0.5:0.5. In addition, it is known that the maximum molar quantity of fuel per unit weight of photocatalyst is obtained for Pd loading weight of 3.90 wt% irrespective of fuel type. It is thought that these results agree with the results shown in Figure 11, Figure 12, Figure 13 and Figure 14.

Figure 11 shows the change in concentration of formed CO with the Pd/TiO_2_ film with the time under the condition of Xe lamp illumination without UV light. In this figure, the impact of molar ratio of CO_2_, H_2_, and H_2_O, as well as Pd loading weight is also presented. Before this experiment, a blank test without Xe lamp illumination had been carried out as a reference, resulting in that no fuel was detected as expected. Table 9 lists the maximum concentration of formed CO under the condition shown in Figure 11.

According to Figure 11 and Table 9, the CO_2_ reduction performance to produce CO is the highest at the molar ratio of CO_2_/H_2_/H_2_O = 1:0.5:0.5 and the maximum concentration of produced fuel is obtained for Pd loading weight of 3.90 wt%. These results are the same as that in the case of illuminating Xe lamp with UV light. The reason why these results are obtained is thought to be the same as explained above in the case of illuminating Xe lamp with UV light. It is found from Figure 11 that the concentration of formed CO is smaller than that under the condition of Xe lamp with UV light. There were no other fuels such as CH_4_, C_2_H_4_, and C_2_H_6_ detected under the condition of Xe lamp illumination without UV light. It is thought that the responsiveness of visible light with Pd/TiO_2_ prepared in this study was too low.

Table 10 shows the maximum molar quantity of CO per unit weight of photocatalyst under the condition of Xe lamp illumination without UV light. The maximum molar quantity of CO per unit weight of photocatalyst is obtained for the molar ratio of CO_2_/H_2_/H_2_O = 1:0.5:0.5 at Pd loading weight of 3.90 wt%. This result is the same as that in the case of illuminating Xe lamp with UV light.

In this study, the maximum molar quantity of CH_4_ per unit weight of photocatalyst is 22.1 μmol/g for the molar ratio of CO_2_/H_2_/H_2_O = 1:0.5:0.5 at Pd loading weight of 3.90 wt% under the condition of Xe lamp illumination with UV light. This maximum value is obtained after 6 h of illumination. According to the previous studies reported, the molar quantities of CH_4_ per unit weight of photocatalyst in the case of CO_2_/H_2_O with Pd/TiO_2_ were 25 μmol/g, 4.8 μmol/g, and 1.9 μmol/g [21,22,40]. These molar quantities of CH_4_ per unit weight of photocatalyst were obtained after 8 [21], 6 [22], and 24 [40] h of illumination, respectively. Another study [23] reported that the molar quantity of CH_4_ per unit weight of photocatalyst in the case of CO_2_/H_2_ with Pd/TiO_2_ was 356 μmol/g which was obtained after 3 h of illumination.

In this study, the maximum molar quantity of CO per unit weight of photocatalyst is 30.3 μmol/g for the molar ratio of CO_2_/H_2_/H_2_O = 1:0.5:0.5 at Pd loading weight of 3.90 wt% under the condition of Xe lamp illumination with UV light. This maximum value is obtained after illumination time of Xe lamp of 6 h. The previous studies reported that the molar quantities of CO per unit weight of photocatalyst in the case of CO_2_/H_2_O with Pd/TiO_2_ were 0.12 μmol/g and 0.13 μmol/g [22,39], while the study reported that the molar quantity of CO per unit weight of photocatalyst in the case of CO_2_/H_2_ with Pd/TiO_2_ was 45 μmol/g [23]. These molar quantities of CO per unit weight of photocatalyst were obtained after illumination of 6 [22], 5 [39], and 3 [23] h, respectively.

Compared to the other studies, CO_2_ reduction performance in terms of producing CH_4_ or CO per unit weight of photocatalyst obtained in this study does not necessarily imply that the photocatalyst was prepared. Additionally, the best time to obtain the highest molar quantity of produced fuel per unit weight of photocatalsyt is almost the same as the previous studies. However, in terms of producing the other fuels such as C_2_H_4_ and C_2_H_6_, which are difficult to produce through CO_2_ reduction and were not reported in the other studies, are confirmed in this study. According to the previous study [21], Pd/TiO_2_ could produce hydrocarbon such as C_2_H_6_ more effectively compared to the other photocatalysts. The CO_2_ molecules activated at Pd sites react with H^+^ and the electrons to produce the intermediate Pd-C=O. Meanwhile, a small amount of CO is generated by C=O desorption, but Pd-C=O further interacts with the dissociated H to form a Pd-C species. Finally, the carbon species generated continue to react with the H species at Pd sites to produce CH_4_. During the CH_4_ formation process, some intermediates (such as ·CH, ·CH_2_, and ·CH_3_) are produced, and C_2_H_6_ is obtained when two ·CH_3_ species interact with each other. Since C_2_H_4_ and C_2_H_6_ have high heating values, producing these fuels have a profound significance in CO_2_ utilization. Therefore, it can be said that this study has realized the photocatalyst having high CO_2_ reduction performance.

Though it is thought that the doped Pd can provide the free electron not only to prevent the recombination of electron and hole produced but also to improve light absorption effect, it is necessary to improve the CO_2_ reduction performance further. This study suggests that different metals should be doped on TiO_2_ to promote the CO_2_ reduction further. The co-doped TiO_2_ such as PbS-Cu/TiO_2_, Cu-Fe/TiO_2_, Cu-Ce/TiO_2_, Cu-Mn/TiO_2_, and Cu-CdS/TiO_2_ were reported to promote the CO_2_ reduction performance of TiO_2_ with H_2_O [4,48]. Then, the promotion of CO_2_ reduction performance by different metal doping is expected when the combination of CO_2_/H_2_/H_2_O is considered. For example, Fe which can absorb the shorter wavelength light than Pd can [48] should be co-used since the amount of light absorbed by the photocatalyst can be increased and an effective utilization of wide range light can be realized by the combination of Fe and Pd.

## 3. Materials and Method

### 3.1. Preparation of Pd/TiO_2_ Photocatalyst

The TiO_2_ film used in this study was prepared using the sol-gel and dip-coating procedure [24,49,50]. At first, [(CH_3_)_2_CHO]_4_Ti (95 wt% purification, produced by Nacalai Tesque Co., Kyoto, Japan) of 0.3 mol, anhydrous C_2_H_5_OH (99.5 wt% purification, produced by Nacalai Tesque Co.) of 2.4 mol, distilled water of 0.3 mol, and HCl (35 wt% purification, produced by Nacalai Tesque Co.) of 0.07 mol were mixed to make the TiO_2_ sol solution. As the basis to coat TiO_2_ film, the sheet of netlike glass fiber was cut into a disc shape whose diameter and thickness were 50 mm and 1 mm, respectively. The disc shaped netlike glass fiber was then immersed into the TiO_2_ sol solution at a speed of 1.5 mm/s and lifted at 0.22 mm/s. The disc was dried and heated at the controlling firing temperature (*FT*) and the firing duration time (*FD*) of 623 K and 180 s, respectively. After the TiO_2_ film was coated on netlike glass disc, the pulse arc plasma method was selected to load Pd on the TiO_2_ film. The pulse arc plasma gun device (ARL-300, produced by ULVAC, Inc., Suzuka, Japan) with Pd electrode having a diameter of 10 mm was used in this study. The quantity of loaded Pd was controlled by pulse number. In this study, the pulse number was varied from 100 to 500, and Pd loading weight with TiO_2_ was measured by EPMA, for each pulse number. It is confirmed that the Pd/TiO_2_ film prepared in this way could not be removed from the netlike glass fiber by rubbing. Figure 12 shows the photos of netlike glass disc before and after coating of Pd/TiO_2_. Since the sheet of netlike glass disc does not have a scouring structure inside it, the TiO_2_ film is coated on the surface of netlike glass fiber and Pd can be deposited on TiO_2_ film by pulse arc plasma method.

### 3.2. Characterization of Pd/TiO_2_ Film

The structural and crystal characteristics of Pd/TiO_2_ film prepared were evaluated by using SEM (JXA-8530F, produced by JEOL Ltd., Tokyo, Japan), EPMA (JXA-8530F, produced by JEOL Ltd., Tokyo, Japan), and EELS (JEM-ARM2007 Cold, produced by JEOL Ltd., Tokyo, Japan). In order to analyze the sample by these equipments, carbon was coated on Pd/TiO_2_ whose thickness was approximately 15 nm by the dedicated device (JEC-1600, produced by JEOL Ltd.) before analysis. This carbon coating was conducted for analysis, while the CO_2_ reduction experiment was carried out without carbon coating. The carbon coating was not conducted for the right photo in Figure 1.

The electron was emitted on the sample by the electron probe applying the acceleration voltage of 15 kV and the current at 3.0 × 10^−8^ A to analyze the surface structure of the sample by SEM. Simultaneously, EPMA detects the characteristic X-ray. The space resolutions for SEM and EPMA were set at 10 μm. The state of prepared photocatalyst, as well as the quantity of doped metal within TiO_2_ film could be known by EPMA analysis.

The electron probe emits electrons to the sample at the acceleration voltage of 200 kV, when the inner structure of the sample is analyzed by STEM. The size, thickness, and structure of loaded Pd were evaluated. The X-ray characteristics of the sample is detected by EDS at the same time. Therefore, the concentration distribution of chemical elements toward thickness direction of the sample is known. In the present paper, the concentration distribution of Ti, Pd, and Si were analyzed.

EELS is used to detect elements, as well as to determine oxidation states of transition metals. The EELS characterization was determined by JEM-ARM200F equipped with GIF Quantum having 2048 ch. The dispersion of 0.5 eV/ch for the full width at half maximum of the zero loss peak was achieved in the study.

### 3.3. CO_2_ Reduction Experiment

Figure 13 shows the experimental setup of the reactor composed of a stainless tube (height of 100 mm and inside diameter of 50 mm), Pd/TiO_2_ film coated on netlike glass disc (diameter of 50 mm and thickness of 1 mm) located on the teflon cylinder (height of 50 mm and diameter of 50 mm), a quartz glass disc (diameter of 84 mm and thickness of 10 mm), an edge cut filter cutting off the light whose wavelength is below 400 nm (SCF-49.5C-42L, produced by SIGMA KOKI CO. LTD., Tokyo, Japan), a 150 W Xe lamp (L2175, produced by Hamamatsu Photonics K. K.), mass flow controller, gas cylinder of CO_2_ and H_2_.

**Figure 15:**
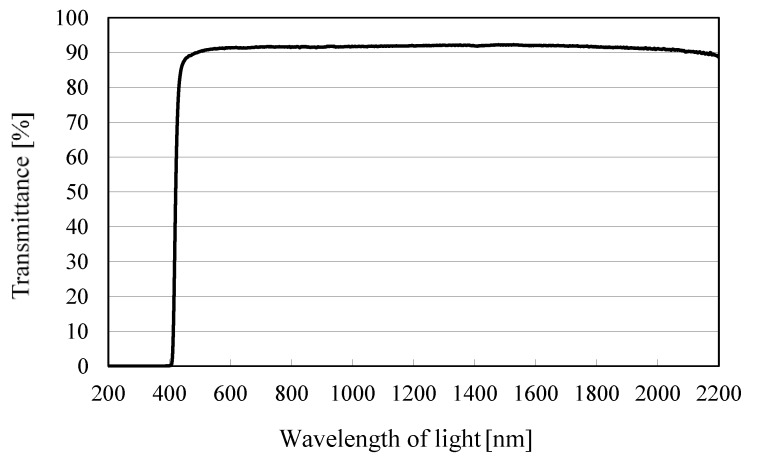
Characterization of edge cut filter to cut off the wavelength of light under 400 nm [49,50].

The reactor volume available for CO_2_ is 1.25 × 10^−4^ m^3^. The light of Xe lamp which is located outside the stainless tube illuminates Pd/TiO_2_ film coated on the netlike glass disc through the edge cut filter and the quartz glass disc that are at the top of the stainless tube. The wavelength of light illuminating by Xe lamp is distributed from 185 nm to 2000 nm. Since an edge cut filter can remove UV components of the light from the Xe lamp, the wavelength from Xe lamp is distributed from 401 to 2000 nm with the filter. Figure 14 shows the spectra data on light intensity of Xe lamp without the edge filter according to the catalog of Xe lamp company. Figure 15 shows the performance of the edge cut filter to cut off the wavelength of light whose wavelength is below 400 nm. The average light intensities of Xe lamp without and with the edge cut filter are 65.0 W/cm^2^ and 40.5 W/cm^2^, respectively.

CO_2_ gas and H_2_ gas whose purity were 99.995 vol% and 99.99999 vol%, respectively were controlled by mass flow controller and mixed in the buffer chamber before the experiment. The mixing ratio of CO_2_ and H_2_ was checked and confirmed by TCD gas chromatograph (Micro GC CP4900, produced by GL Science, Tokyo, Japan) before being introduced into the reactor. The distilled water was then injected into the reactor via gas sampling tap and when Xe lamp was turned on. The water was injected and vaporized by the heat of Xe lamp completely. The molar ratio of CO_2_/H_2_/H_2_O was set at 1:0.5:0.5, 1:0.5:1, 1:1:0.5, 1:1:1, 1:2:2. The temperature in reactor rose up to 343 K within 1 h and was kept at about 343 K during the entire experiment.

In the CO_2_ reduction experiment with UV light, samples of the gas in the reactor were taken every 6 h, while in the CO_2_ reduction experiment without UV light samples were taken every 24 h due to the difference of reaction speed of prepared photocatalyst under these two conditions. The gas samples were analyzed using FID gas chromatograph (GC353B, produced by GL Science) and methanizer (MT221, produced by GL Science). FID gas chromatograph and metanizer can be analyzed in the minimum range of 1 ppmV.

## 4. Conclusions

The following conclusions could be drawn from this study:

- The nanosized Pd particles could be loaded on TiO_2_ uniformly by the pulse arc plasma method. Pd in Pd/TiO_2_ prepared by this method exists in the form of Pd metal.
- The highest CO_2_ reduction performance to produce CO, CH_4_, C_2_H_4_, and C_2_H_6_ was obtained at the molar ratio of CO_2_/H_2_/H_2_O = 1:0.5:0.5 with Xe lamp illumination with or without UV light. It is revealed that the molar ratio of CO_2_/total reductants = 1:1 is the optimum to produce fuels.
- The maximum molar quantity of fuel per unit weight of photocatalyst is obtained at Pd loading weight of 3.90 wt% irrespective of fuel type. In this study, the maximum molar quantities of CO and CH_4_ per unit weight of photocatalyst were 30.3 μmol/g and 22.1 μmol/g, respectively, for the molar ratio of CO_2_/H_2_/H_2_O = 1:0.5:0.5 at Pd loading weight of 3.90 wt% under the condition of Xe lamp illumination with UV light.
- The Pd/TiO_2_ photocatalyst prepared in this study could produce C_2_H_4_ and C_2_H_6_, as well as CO and CH_4_, therefore, it can be said that the photocatalyst prepared in this study has realized to have the higher CO_2_ reduction performance.

## Figures and Tables

**Table 1:** Comparison of maximum concentration of formed CO with illumination time among different molar ratios of CO_2_/H_2_/H_2_O and Pd loading weight with UV light illumination.

	1:0.5:0.5	1:0.5:1	1:1:0.5	1:1:1	1:2:2
0.44 wt%	91 ppmV	80 ppmV	63 ppmV	45 ppmV	18 ppmV
3.90 wt%	313 ppmV	268 ppmV	193 ppmV	171 ppmV	109 ppmV
4.97 wt%	107 ppmV	66 ppmV	66 ppmV	56 ppmV	51 ppmV

**Table 2:** Comparison of maximum concentration of formed CH_4_ with illumination time among different molar ratios of CO_2_/H_2_/H_2_O and Pd loading weight with UV light illumination.

	1:0.5:0.5	1:0.5:1	1:1:0.5	1:1:1	1:2:2
0.44 wt%	143 ppmV	72 ppmV	123 ppmV	31 ppmV	0 ppmV
3.90 wt%	227 ppmV	166 ppmV	121 ppmV	134 ppmV	85 ppmV
4.97 wt%	211 ppmV	113 ppmV	113 ppmV	108 ppmV	48 ppmV

**Table 3:** Comparison of maximum concentration of formed C_2_H_4_ with illumination time among different molar ratios of CO_2_/H_2_/H_2_O and Pd loading weight with UV light illumination.

	1:0.5:0.5	1:0.5:1	1:1:0.5	1:1:1	1:2:2
0.44 wt%	0 ppmV	0 ppmV	0 ppmV	0 ppmV	0 ppmV
3.90 wt%	28 ppmV	20 ppmV	15 ppmV	0 ppmV	0 ppmV
4.97 wt%	0 ppmV	0 ppmV	0 ppmV	0 ppmV	0 ppmV

**Table 4:** Comparison of maximum concentration of formed C_2_H_6_ with illumination time among different molar ratios of CO_2_/H_2_/H_2_O and Pd loading weight with UV light illumination.

	1:0.5:0.5	1:0.5:1	1:1:0.5	1:1:1	1:2:2
0.44 wt%	0 ppmV	0 ppmV	0 ppmV	0 ppmV	0 ppmV
3.90 wt%	18 ppmV	9 ppmV	0 ppmV	0 ppmV	0 ppmV
4.97 wt%	0 ppmV	0 ppmV	0 ppmV	0 ppmV	0 ppmV

**Table 5:** Comparison of the maximum molar quantity of CO per unit weight of photocatalyst with illumination time among different molar ratios of CO_2_/H_2_/H_2_O and Pd loading weight with UV light illumination (unit: µmol/g).

	1:0.5:0.5	1:0.5:1	1:1:0.5	1:1:1	1:2:2
0.44 wt%	9.27	8.18	9.06	4.63	1.81
3.90 wt%	30.4	26.0	18.9	16.6	10.6
4.97 wt%	5.97	3.66	3.52	3.10	2.84

**Table 6:** Comparison of the maximum molar quantity of CH_4_ per unit weight of photocatalyst with illumination time among different molar ratios of CO_2_/H_2_/H_2_O and Pd loading weight with UV light illumination (unit: µmol/g).

	1:0.5:0.5	1:0.5:1	1:1:0.5	1:1:1	1:2:2
0.44 wt%	14.6	7.33	6.91	3.20	0
3.90 wt%	22.1	16.1	11.8	13.1	8.25
4.97 wt%	11.8	6.28	6.85	6.04	2.69

**Table 7:** Comparison of the maximum molar quantity of C_2_H_4_ per unit weight of photocatalyst with illumination time among different molar ratios of CO_2_/H_2_/H_2_O and Pd loading weight with UV light illumination (unit: µmol/g).

	1:0.5:0.5	1:0.5:1	1:1:0.5	1:1:1	1:2:2
0.44 wt%	0	0	0	0	0
3.90 wt%	2.69	1.91	1.46	0	0
4.97 wt%	0	0	0	0	0

**Table 8:** Comparison of the maximum molar quantity of C_2_H_6_ per unit weight of photocatalyst with illumination time among different molar ratios of CO_2_/H_2_/H_2_O and Pd loading weight with UV light illumination (unit: µmol/g).

	1:0.5:0.5	1:0.5:1	1:1:0.5	1:1:1	1:2:2
0.44 wt%	0	0	0	0	0
3.90 wt%	1.75	0.91	0	0	0
4.97 wt%	0	0	0	0	0

**Table 9:** Comparison of maximum concentration of formed CO with illumination time among different molar ratios of CO_2_/H_2_/H_2_O and Pd loading weight without UV light illumination.

	1:0.5:0.5	1:0.5:1	1:1:0.5	1:1:1	1:2:2
0.44 wt%	42 ppmV	37 ppmV	35 ppmV	35 ppmV	28 ppmV
3.90 wt%	67 ppmV	57 ppmV	48 ppmV	42 ppmV	33 ppmV
4.97 wt%	53 ppmV	40 ppmV	48 ppmV	38 ppmV	31 ppmV

**Table 10:** Comparison of maximum molar quantity of CO per unit weight of photocatalyst with illumination time among different molar ratios of CO_2_/H_2_/H_2_O and Pd loading weight without UV light illumination (unit: µmol/g).

	1:0.5:0.5	1:0.5:1	1:1:0.5	1:1:1	1:2:2
0.44 wt%	3.97	3.58	3.34	3.37	2.63
3.90 wt%	6.34	5.42	4.64	4.03	3.21
4.97 wt%	2.77	2.11	2.56	2.06	1.63
